# Multi-channel magnetic resonance spectroscopy graphical user interface (McMRSGUI)

**DOI:** 10.1371/journal.pone.0299142

**Published:** 2024-02-28

**Authors:** Travis Carrell, Mary P. McDougall

**Affiliations:** Department of Biomedical Engineering, Texas A&M University, College Station, Texas, United States of America; Museo Storico della Fisica e Centro Studi e Ricerche Enrico Fermi, ITALY

## Abstract

This work introduces an open-sourced graphical user interface (GUI) software enabling the combination of multi-channel magnetic resonance spectroscopy data with different literature-based methods for the improvement of the quality and reliability of combined spectra. The multi-channel magnetic resonance spectroscopy graphical user interface (McMRSGUI) is a MATLAB-based spectroscopy processing GUI equipped to load multi-channel MRS data, pre-process, combine, and export combined data for evaluation with open-source quantification software (jMRUI). A literature-based, decision-tree process was incorporated into the combination type selection to serve as a guide to minimize spectral distortion in selecting between weighting methods. Multi-channel, simulated spectra were combined with the different combination techniques and evaluated for spectral distortion to validate the code. The incorporation of the combination methods into a single processing software enables multi-channel magnetic resonance spectroscopy (MRS) data to be combined and compared for improved spectral quality with little user knowledge of combination techniques. Through the spectral peak distortion simulation of the combination methods, combined signal-to-noise ratio (SNR) values from the literature were verified. The spectral peak distortion simulation provides a secondary tool for researchers to estimate the spectral SNR levels when spectral distortion could occur and use this knowledge to further guide the selection of their combination technique. The McMRSGUI provides a software toolkit for evaluating multi-channel MRS data and their combination. Simulations evaluating spectral distortion at different noise levels were performed for each combination method to validate the GUI and demonstrate a method for researchers to assess the combined SNR levels at which they could be introducing spectral distortion.

## Introduction

Magnetic resonance spectroscopy is a noninvasive method that provides access to important biochemical information from biological pathways and diseases in the body. Challenges associated with MRS of all nuclei involve detecting small concentrations, varying natural abundance and sensitivities of different nuclei relating to the overall obtainable signal intensity, and the balance between feasible scan times and appropriate localization.

One of the methods for alleviating some of these problems involves array coils and their increased sensitivity over a larger field of view. Roemer’s seminal work described how an array of coils could be optimally combined with complex weighting factors for an improved result using the B_1_^-^ receive fields and the noise covariance [1], but obtaining the requisite information required for the formulation isn’t necessarily feasibly determined within a clinical setting. The “optimal” method for combining experimental spectroscopy data from multiple channels has been an active topic of discussion within literature [2–4], resulting in multiple combination techniques [2–9]. Some of the techniques require either additional noise scans for obtaining noise correlation or covariance maps [3] or prior unsuppressed water reference scans [2, 4, 5], to obtain weighting factors. Others simply require the SNR or variations of subcomponents of the SNR with regards to a certain metabolite [2, 10, 11].

The tradeoff of using more scanner time for acquiring additional data and increasing the degree of post-processing results in either a better understanding of the noise correlation and covariance amongst the channels or an improved estimation of the field maps of the channels through the increased sensitivity of ^1^H data. Regardless of the technique, the end goal of the combination is to improve the spectral SNR for reliability of quantification and fitting without introducing significant bias or spectral distortion. Spectral distortion directly results in inaccurate findings for quantification of metabolites. As the goal for spectroscopy processing is to minimize user interaction, spectral distortion can go unnoticed. Therefore, the selection of the combination technique should be determined based on the available additional clinical data, noise correlation between channels, degree of user interaction for phasing data, and the relative SNR of the combined spectra to ensure that the combined result is as accurate as possible.

Magnetic resonance vendors each have their own processing software, which often performs combination and some pre-processing operations on multi-channel data before allowing access to the “raw” data. Per a recent experts’ consensus report, it is recommended that individual transient data be accessible for preprocessing and radiofrequency (RF) coil combination, as well as effectively communicating all actions performed on the data to increase reproducibility of results [12].

Several groups have put forth open-source software to enable the processing of MRS data from the initial data loading all the way through data quantification [13–18] or simply handle loading and preprocessing of data and transfer processed data to existing programs for quantification [19]. Variations exist among the programs as to the coding language, whether the source code is openly available (OSPREY, OXSA, FSL-MRS, FID-A), if they are packaged in a manner to make the program accessible amongst different computer architectures (jMRUI, INSPECTOR), or they are available via web-based applications (SelectWave). Benefits arise from all three types of systems as modularity, visibility, and flexibility to add or modify code as newer methods become available is a key argument for making the source code available, while providing a packaged program can enable a more sequenced progression, platform-independence, and wider potential use. The combination of both methods provides the greatest potential for usage and research opportunities. Regardless, both methods still require programming that is specific to a certain niche application and will require continued updates as newer processes become available. Therefore, open-source MRS code requires readability, low-entry to understanding, and transparency to be truly effective in an ever-changing field.

This work presents an alternative for pre-processing and combining of MRS data; presenting users with literature-based options to best combine and phase their multi-channel data in a format that can be presented as a standalone program or be accessible via a coding language commonly used by those in the field. It specifically addresses the gap amongst open-source MRS codes concerning access to different coil combination techniques to reduce the introduction of bias via the combination method. Furthermore, an additional simulation is presented that provides utility in identifying SNR levels in which bias can be introduced from the combination technique. Although it was designed specifically for use with single voxel, x-nuclei data, the toolkit also incorporates methods that can be utilized for ^1^H spectra.

## Methods

The McMRSGUI was developed in MATLAB® (Mathworks, Natick, MA) using App Designer to allow the graphical user interface to be published in a stand-alone capacity for utilization on Windows-based systems or be used within MATLAB’s coding environment for research use. This platform enables flexibility as the template code can be adjusted to specific research needs and then be compiled and shared with fellow researchers. The compiled GUI can be run on any Windows-based computer that has the MATLAB Runtime environment installed, which is free and available for download from the MathWorks website [20].

### Data loading

The program is configured to load time domain spectroscopy data within three file types: Varian (.fid), jMRUI Data Textfile (.txt), or a MATLAB structure (.mat). Time domain data must represent baseband or digitally demodulated spectroscopy data. Necessary information for loaded files includes the complex free induction decay (FID) data, center frequency, and spectral bandwidth. Multiple files or acquisition sets can be loaded at once and will be treated as if they are a continuation of the previously loaded file(s) acquisitions.

### Preprocessing

Functionality for preprocessing of individual channel data, such as averaging transients with a sliding window average of user-defined length for studies requiring temporal resolution, manual 0-order and 1^st^-order phasing, baseline correction [21], zero-padding, subtraction of another file for removal of the water peak or broad baseline macromolecules, and different viewing configurations either in the time domain or frequency domain, are built into the program. Additionally, line broadening is included for data viewing but is not recommended for data quantification. Inspection of the data for data quality can be performed through viewing the FID, noise-normalized spectra, or a waterfall plot. Spectral evaluation of linewidth and SNR are performed using the maximum peak or on a user-defined frequency region. Noise selection limits and frequency axis viewing limits are manually set by the user. Fully preprocessed data can be exported in two different formats: either in the.mat structure “Processed” or in the jMRUI Data Textfile format. Both formats can be reloaded and preprocessed as necessary.

### Multi-channel combination

**Fig 1:**
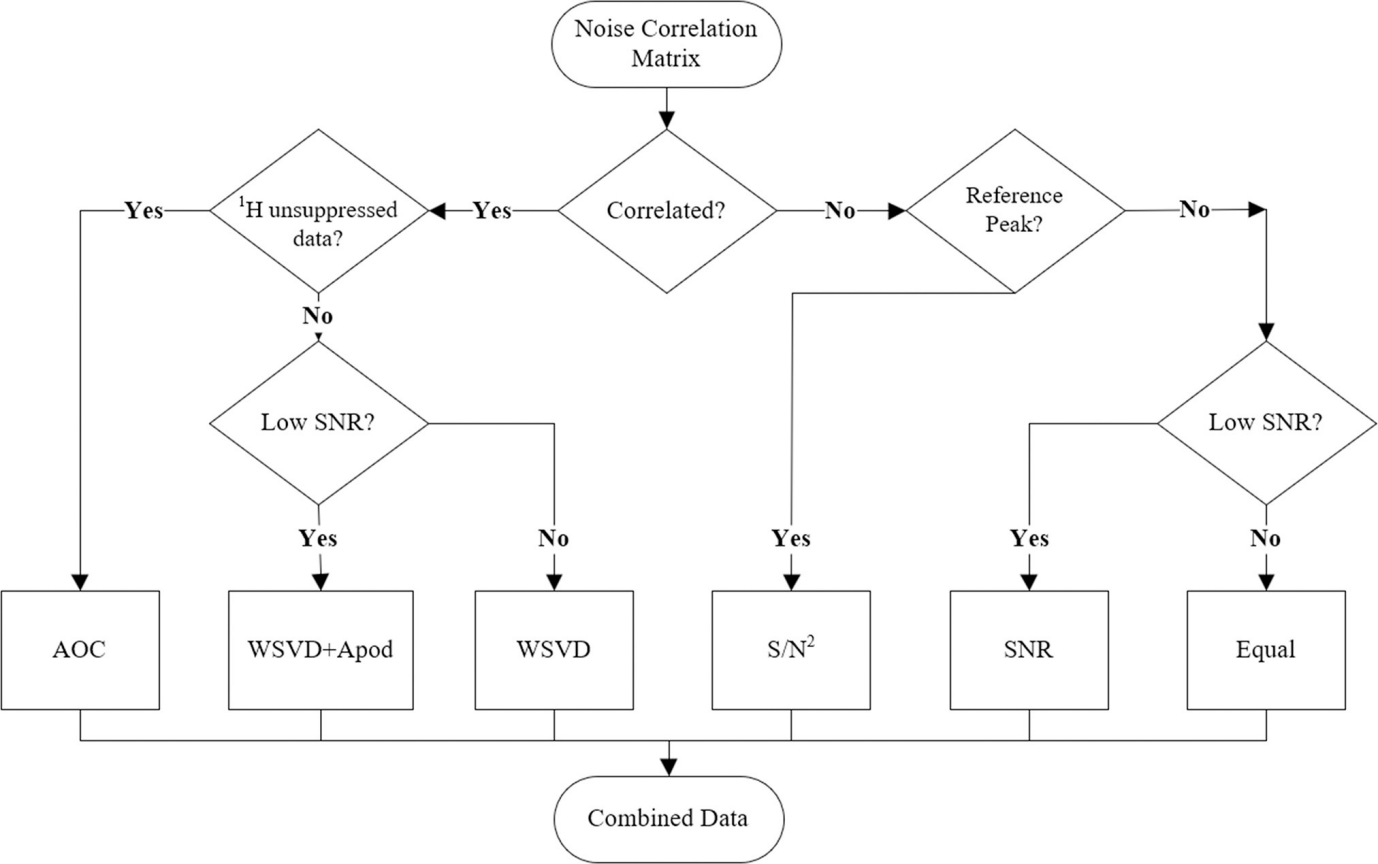
Flowchart of the decision tree for multi-channel spectroscopy combination. Multi-channel spectroscopy combination method decision tree logic utilized within the McMRSGUI. The user has the option to specifically select a method or use the program as a guide for selecting an appropriate combination method. Utilization of supplemental ^1^H unsuppressed water data for determining weighting factors improves the accuracy of the combination method(s) in scenarios of low SNR combination. The weighting methods are arranged from left to right in order of literature-recommended order for the scenario of correlation between channels.

The preprocessed multi-channel data is combined in the spectral domain by multiplying each channel by a weighting factor before summation with the other weighted channel data. Selection of the combination method can be chosen directly by the user or the decision tree matrix, shown in Fig 1, will assist the user in selecting an appropriate method. Six weighting options are available within the program: equal weighting, SNR-weighting [9, 10], S/N^2^-weighting [2], whitened value singular-value decomposition (WSVD) with or without apodization (Apod) [3, 22], and adaptively optimized combination (AOC) [4]. The first three methods assume that the noise between channels is not correlated [4]. Phasing of the spectra is necessary to adequately estimate the signal and noise components for the first two methods, while the S/N^2^ method utilizes complex weighting factors obtained from the fitting of a reference peak within the data [2]. Additionally, the alignment of peak frequencies is an option afforded to the user to correct for frequency shifts amongst the channels due to positioning of the array for the equal and SNR weighing methods. The WSVD method requires the calculation of a noise covariance matrix obtained from a user-defined noise free region in the spectral domain or a separate noise scan obtained with the transmitter turned off [3]. In scenarios where the SNR of the data is low, utilizing the WSVD+Apod method can improve the estimation of the weighting factors through the application of linebroadening or apodization. The quality of the estimate of the WSVD weighting factors displays in a range from 0 to 1, with 1 representing the highest quality estimation [22]. The AOC method incorporates an unsuppressed water scan into the weighting factors and the inversion of the full noise correlation matrix [4]. The full noise correlation matrix incorporates the coil covariances onto the diagonal components of the noise correlation, enabling this method to better estimate the influences from intrinsic and extrinsic noise sources. Like the WSVD method, the quality of the full noise correlation is determined by the number of data points used to calculate the correlation matrix.

To validate the McMRSGUI processing code, two Lorentzian resonance peaks were modeled according to the simulation by Wu et al. for measuring distortion introduced by multi-channel combination techniques [4]. The Lorentzian peaks were modeled according to Eq (1),

$$x(p)=\sum_{q=1}^{2}a_{q}e^{j\theta_{q}}\;e^{(-d_{q}+j2\pi f_{q})\frac{p}{f_{s}}}$$ (1)

where *f*_*s*_ is the sampling frequency, and *a*_*q*_, *θ*_*q*_, *d*_*q*_, and *f*_*q*_ denote the amplitude, phase, damping factor, and resonance frequency of the *q*th sinusoid, respectively. Parameters used for the simulation and the associated scaling of the eight generated coils elements are in Table 1. Correlated noise between channels was simulated utilizing the full noise correlation matrix with values approximating Wu et al.’s simulation [4]. Distortion was measured as the difference between the ratio of the areas underneath the two peaks from the original model spectrum and the combined spectrum. jMRUI and the AMARES algorithm were utilized for calculating the areas under both peaks following data combination [17, 23].

**Table 1:** Parameters for Lorentzian peak simulation and coil element scaling factors.

Peak q			a_q_			Θ_q_/rad		d_q_			f_q_/Hz	
1 (creatine, CH_3_)			15			0		10			132	
2 (creatine, CH_2_)			15			0		10			56	
**Coil Element Scaling Factors**												
Channel	1	2		3	4		5		6	7		8
Scale Factor	1	0.8		0.7	0.6		0.5		0.65	0.9		0.93

For the simulation, the noise content was varied between levels corresponding to high SNRs and low SNRs, where the SNR was defined as the signal amplitude of the highest peak over the standard deviation of the noise after the application of a matched filter [24]. The noise region was selected as the last 400 points of the simulated spectra where there were no peaks. As each combination method produced a different SNR for the same added noise level, the noise level was used as the common variable to compare the overall distortion level between the methods. Monte Carlo simulations were utilized to generate 300 variations of the eight-element noise data for each noise level utilizing MATLAB’s white gaussian noise function with a 50 Ohm impedance. The results of the Monte Carlo simulations enabled the comparison of the mean and standard deviation for each noise level and associated combination method. The assumption that the AOC method could adequately determine the coil sensitivities and spatial phase shifts of the individual coil elements from an unsuppressed water resonance was utilized, as per Wu et al.’s simulation [4]. The S/N^2^ method’s signal amplitude factors were calculated using the AMARES algorithm in jMRUI [17, 23].

## Results

**Fig 2:**
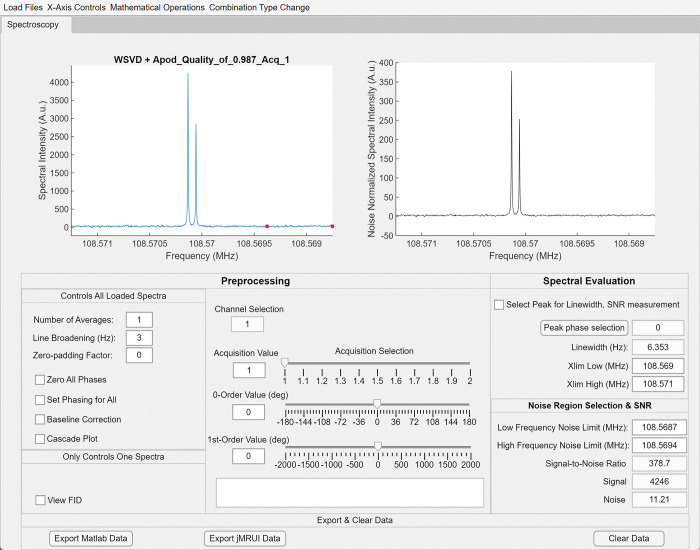
Front panel of McMRSGUI. McMRSGUI front panel showing the first acquisition of the WSVD+Apod combined spectra from the simulated dataset with 37.5 dB of noise added. The preprocessing controls are shown in the leftmost tab group and the spectral evaluation tools are shown on the right. The quality of the WSVD+Apod fitting is shown with the title above the spectrum.

The fully constructed MATLAB GUI for the McMRSGUI is shown in Fig 2. The WSVD+Apod combined data of the simulated data case with 37.5 decibel (dB) of noise added is shown as an example to illustrate the functionality of some of the preprocessing and spectral evaluation tools. Further examples of the functionality and utilization of the program can be found in the Supporting Information Materials.

**Fig 3:**
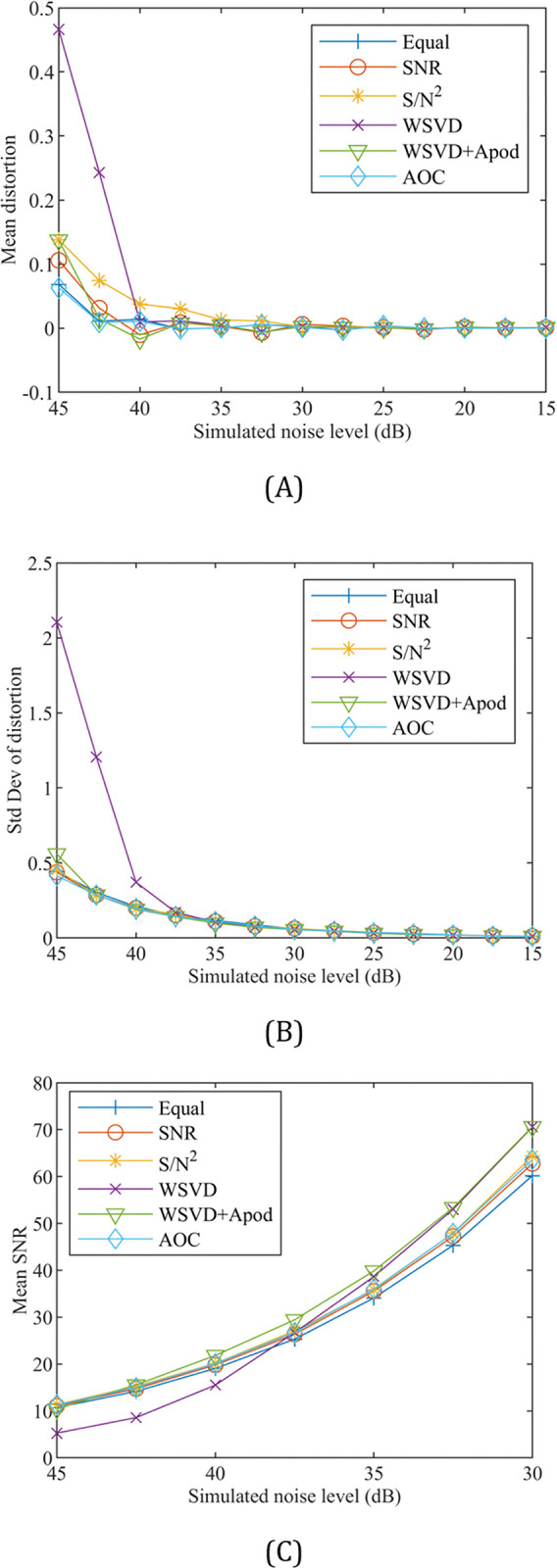
Mean distortion, standard deviation of distortion, and mean SNR plots as a function of the simulated noise for the combination methods. (A) The mean distortion of the combined spectrums’ peaks ratio resulting from the Monte Carlo simulation. Due to the signal content remaining constant and the associated SNR of the combined spectrum varying due to the different combination methods, the mean distortion is shown as a function of the simulated noise level. (B) The standard deviation of the distortion from the true ratio is also shown as a function of the simulated noise level. (C) The mean SNR of each combination derived from the highest peak’s amplitude over the standard deviation of the noise in the range of the highest simulated noise levels.

Fig 3A and 3B show the mean and standard deviation of the combined spectrum’s ratio of the area of the two peaks from that of the original model spectrum following Monte Carlo simulations at different noise levels. The corresponding mean SNR of each method is shown in Fig 3C. At lower added noise levels, corresponding to higher SNR levels, all the methods combine the spectrum with little to no mean distortion. Increasing levels of distortion occur as higher levels of noise are added, making the signal more difficult to distinguish from the noise. The WSVD+Apod method outperforms the original WSVD method at lower SNR levels due to its reduction in high frequency noise from linebroadening when estimating the weighting factors. Overall, the AOC method had the lowest or one of the lower levels of distortion for the tested noise ranges. All of the above results represent a similar performance to the simulation completed by Wu et al. [4], validating that the combination methods are accurately implemented. Due to the simulation being run with no phase distortion on the channels, this represented the best-case scenario and mitigated the requirement to manually phase the spectra for the equal and SNR methods. This resulted in an improved performance for these methods at lower SNR values than would normally be expected, as these methods rely on manual phasing or automatic phasing algorithms before the calculation of the scalar weights.

## Discussion

For multi-channel MRS data, the selection of the combination type has implications for the quality and relative distortion from the absolute spectra that is obtainable. As the absolute result cannot be adequately inferred from the limited information obtained within a clinical setting, the best estimation represents the highest achievable standard for MRS combination. The estimations for all the combination methods converge to a similar result for cases where the SNR is adequately high. As high SNR is rarely the case for clinical spectroscopy due to the need for localization, limited scan times, physiological concentrations, and decreased sensitivity for x-nuclei, the selection choice for the combination of multi-channel data becomes weightier. The methods that utilize an unsuppressed water scan benefit from the much higher sensitivity within the body for determining phases and scaling factors. As x-nuclei studies do not have the luxury of the higher sensitivity from water, a prominent metabolite, such as phosphocreatine (PCr) for ^31^P, can be used. Solely utilizing the water content or prominent metabolite for determining the scaling coefficients disregards the influence of the noise correlation between channels. Ideally, the receive array being used would have little to no correlation between channels due to good design, sufficient decoupling from preamplifiers, and no development of eddy current signals from the patient or surrounding MR components. In this case, the noise correlation matrix could be disregarded and one of the SNR-based methods could be used. With the advent and use of higher channel count arrays, correlation between channels will undoubtedly increase. Therefore, an understanding of the noise correlation should be factored into the selection for combination method if possible.

In their work, Rodgers et al. provided an SNR guideline of 35, 15, and 60 at which the WSVD, WSVD+Apod, and S/N^2^ methods, respectively, began to produce higher SNR values than what Rodgers et al. called Roemer’s (BS B_1_^-^) combination [3]. To verify the validity of this threshold, a 3 Hz matched filter was applied to the output of the combined spectra and found that the WSVD, WSVD+Apod, and S/N^2^ methods produce similar SNR values to those reported by Rodgers et al. at simulated noise levels of ~35 dBm, ~42.5 dBm and ~30 dBm, respectively. These simulated noise levels were all above the point at which the combination methods began to introduce distortions into the combined spectrum in Fig 3, thus supporting the conclusion by Rodgers et al.

**Fig 4:**
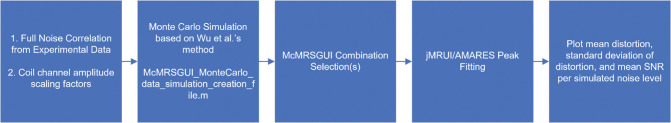
Simulation block diagram for distortion and bias testing via combination techniques. Block diagram showing the simulation process and associated MATLAB scripts/GUI for calculating the SNR levels at which bias and distortion can be introduced by the combination techniques.

An extension of the above validation can be applied by researchers to their in vivo data sets by utilizing the same simulation performed in this work, as detailed by Fig 4. The full noise correlation matrix and approximate coil amplitudes from the in vivo data can be inputted into the simulation for spectral distortion proposed by Wu et al. [4]. The addition of noise levels, as performed in this paper, can then be run through Monte Carlo simulations to verify when the ratio of peak areas of the combined spectrum departs from the absolute ratio, providing validation for the SNR at which the clinical data were combined with regards to distortion and the combination selection. This can provide researchers with a method to mitigate distortion in their data as well as increase the SNR and associated confidence in the fitting of the metabolites within their data.

## Conclusion

The McMRSGUI addresses a gap within open-source MR spectroscopy codes as it consolidates multiple literature-based, multi-channel combination techniques within a single, flexible GUI for utilization in determining the proper technique based on the available additional scan information, noise correlation between channels, and combined SNR. The decision tree provides a simplified approach for the selection of the various techniques. Data can then be exported for quantification with other open-source software. This software was validated using a spectral peak distortion simulation that both verified SNR values presented by Rodgers et al. [3] and provides insight into the point at which distortion is likely to be introduced due to the combination method. In addition to the decision tree, this simulation can be utilized by researchers to reduce the likelihood that the chosen combination technique will introduce distortion in their multi-channel MRS data.
